# When viral myocarditis meets thrombosis tendency: deep analysis of a complex case report

**DOI:** 10.3389/fcvm.2025.1641074

**Published:** 2025-08-20

**Authors:** Qian Ding, Yun Zhang, Dongbei Li, Wenhua Liu, Jing Feng, Shuang Li, Wei Chen, Mu Guo

**Affiliations:** ^1^ Department of Chronic Disease Management, Shanghai Fourth People’s Hospital, School of Medicine, Tongji University, Shanghai, China; ^2^ Department of Cardiology, Shanghai Fourth People’s Hospital, School of Medicine, Tongji University, Shanghai, China

**Keywords:** viral myocarditis, EBV/CMV infection, hereditary thrombophilia, multiorgan embolism, case report

## Abstract

This case report presents a 43-year-old male patient with severe symptoms who was admitted due to dyspnea following physical activity, cough accompanied by fever, lower limb edema, and hemoptysis. The patient had a 20-year history of hypertension. Examinations revealed bilateral lower pulmonary artery thrombosis, a left ventricular thrombus, pulmonary infarction, and reduced left ventricular systolic function, with a lowest left ventricular ejection fraction (LVEF) of 26.5%. Genetic testing indicated the presence of methylenetetrahydrofolate reductase (MTHFR) (C677T) CT type and plasminogen activator inhibitor-1 (PAI-1) (4G/5G) 4G/5G type, while pleural fluid sequencing confirmed Epstein–Barr virus (EBV)/cytomegalovirus (CMV) infection, leading to a diagnosis of viral myocarditis. Treatment included low molecular weight heparin for anticoagulation, glucocorticoids, and measures to improve cardiac function. During treatment, the patient developed a cerebral infarction. Anticoagulation was maintained post-evaluation due to the *PAI-1* mutation and was later adjusted to rivaroxaban. Following treatment, inflammatory markers and coagulation function improved, cardiac function recovered (LVEF increased to 53%), and the thrombus resolved. The combination of EBV/CMV infection with *MTHFR* and *PAI-1* mutations synergistically induced thrombosis through the “virus-inflammation-gene” pathway. This case underscores the importance of early pathogen and genetic screening, as well as personalized anticoagulation strategies, such as substituting warfarin with rivaroxaban. The potential synergistic effect of infection and hereditary thrombophilia in multi-organ embolism warrants careful consideration.

## 1 Introduction

Viral myocarditis is characterized by inflammation of the myocardium due to viral infections, with common pathogens including Epstein–Barr virus (EBV) and cytomegalovirus (CMV) (1, 2). These viral infections not only cause direct damage to myocardial cells but also activate the coagulation system through cytokine storms, particularly involving interleukin-6 (IL-6) and tumor necrosis factor-alpha (TNF-α), which can lead to a hypercoagulable state (3). Additionally, genetic factors, such as mutations in the methylenetetrahydrofolate reductase (MTHFR) and plasminogen activator inhibitor-1 (PAI-1) genes, may further elevate the risk of thrombosis (4, 5), resulting in multi-organ thrombosis. Currently, research on the mechanisms by which viral infections and hypercoagulable states interact to cause multi-organ embolism is limited, particularly regarding the role of genetic susceptibility, which remains not fully understood. Clinical management strategies, including the intensity of anticoagulation and the timing of steroid administration, require further investigation. This case analysis aims to explore the pathophysiological mechanisms, diagnostic and therapeutic challenges, and individualized anticoagulation strategies in viral myocarditis complicated by a hypercoagulable state.

## 2 Case presentation

**Figure 1:**
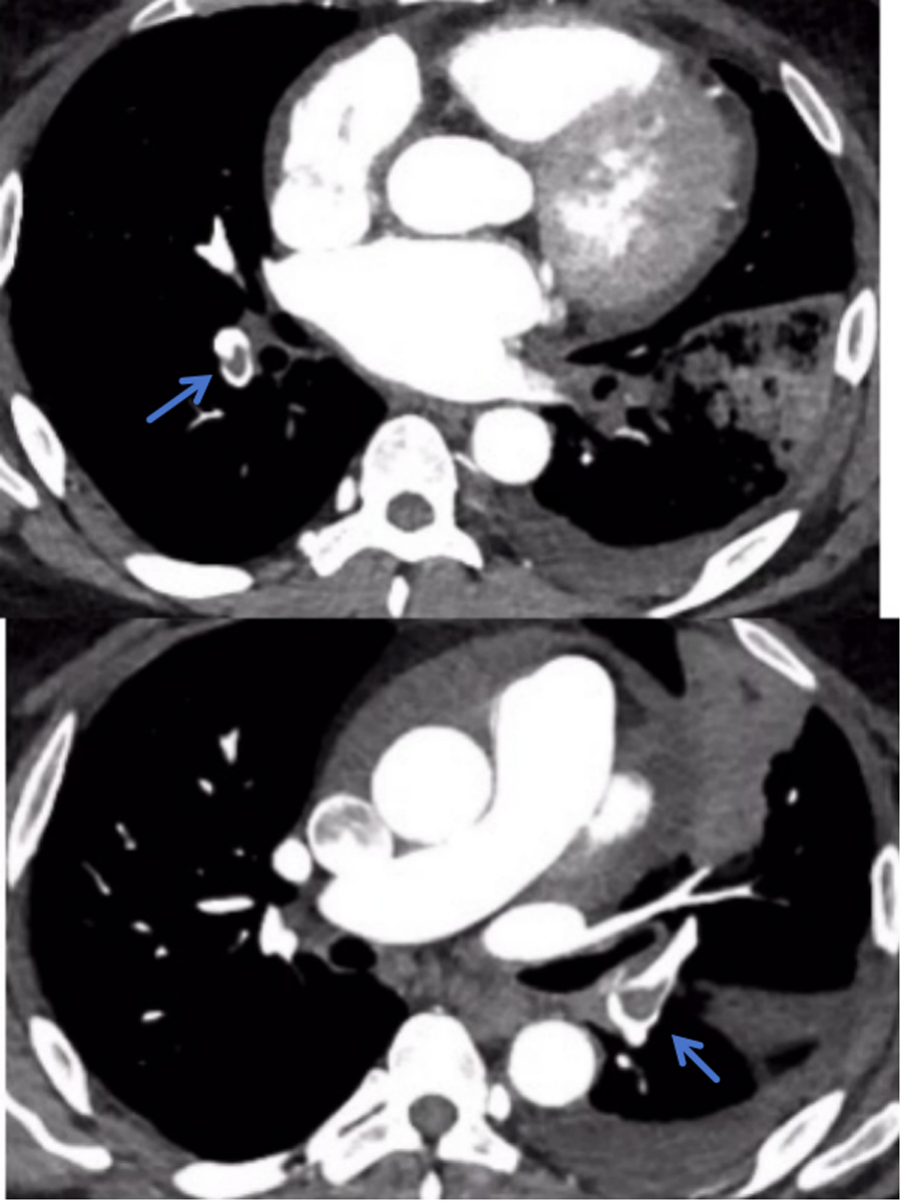
TRO CTA showed bilateral lower pulmonary artery/branch thrombi.

A 43-year-old male (98 kg) presented with 1-month history of exertional dyspnea, 2-week cough, fever, lower limb edema, and 2-day hemoptysis, with no family history of hereditary diseases. The patient had not received treatment elsewhere prior to admission. Vital signs showed temperature 37–38°C, respiratory rate 35 breaths/min, SpO_2_ 98% (air), heart rate 123 bpm, blood pressure 139/97 mmHg, and lip cyanosis. Physical exam revealed jugular vein distension, positive hepatojugular reflux, bilateral coarse breath sounds with left lower lobe moist rales, regular heart rhythm without murmurs, distended abdomen with liver palpable 3 cm below costal margin, and severe facial/lower limb edema. With 20-year hypertension history (no family heart/thrombotic disease), admission triple-rule-out computed tomography angiography (TRO CTA) for chest pain showed bilateral lower pulmonary artery/branch thrombi (Figure 1). Antiphospholipid antibodies, influenza A/B, and SARS-CoV-2 tests were negative (Table 1).

**Table 1:** Changes in key indicators before and after treatment.

Test items	Pre-treatment	Post-treatment	Clinical change and interpretation
White blood cell count.	21.18 × 10^9^/L	9.01 × 10^9^/L	Alleviation of inflammation
Procalcitonin	1.933 ng/ml	0.047 ng/ml	Effective resolution of inflammatory response
C-Reactive Protein	220.86 mg/L	3.73 mg/L	Reduction in inflammation
IL-6	609.7 pg/ml	4.69 pg/ml	Alleviation of inflammation
D-Dimer	8.08 mg/L	0.66 mg/L	Improvement in secondary hyperfibrinolysis, reduced thrombosis risk
Fibrinogen	5.75 g/L	4.37 g/L	Gradual normalization of coagulation function
Prothrombin Time	22.3 s	16.6 s	Improvement in coagulation function
Troponin I	0.336 ng/ml	0.275 ng/ml	Gradual repair of myocardial injury
Myoglobin	415.30 ng/ml	53.96 ng/ml	Gradual improvement in myocardial function
CK-MB	3.61 ng/ml	1.99 ng/ml	Improvement in myocardial function
NT-proBNP	1,079 pg/ml	1,124.00 pg/ml	Possible delayed BNP decline during early cardiac function recovery
Creatinine	112 μmol/L	84.5 μmol/L	Gradual decline indicating renal function recovery
Serum Potassium	5.84 mmol/L	4.29 mmol/L	Gradual return to normal
IVS Thickness	15 mm	11 mm	Improvement in cardiac remodeling
LVEF	43%	53%	Improvement in heart failure

CK-MB, creatine kinase-MB isoenzyme; NT-proBNP, n-terminal pro-B-type natriuretic peptide; IVS, interventricular septum; LVEF, left ventricular ejection fraction; Echo, echocardiography.

**Figure 2:**
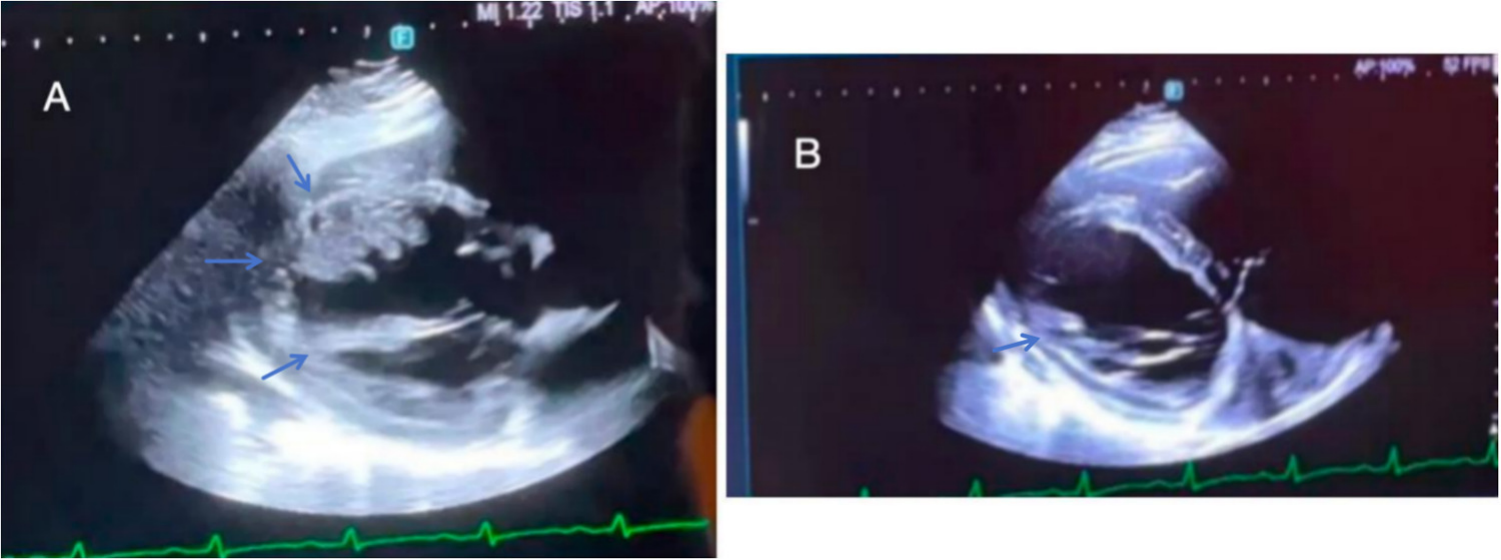
**(A)** On the fourth day of hospitalization, UCG revealed biatrial/right ventricular enlargement, left ventricular wall motion abnormalities, reduced LVEF (42%), mild left ventricular wall thickening (4 mm), intraventricular thrombus, and pericardial effusion. **(B)** On the thirteenth day of hospitalization, UCG revealed an interventricular septum measuring 15 mm, an LVEF of 34%, reduced left ventricular thrombus and normal right heart function.

On the fourth day of hospitalization, sputum culture showed no bacterial/fungal growth, with improved inflammatory/myocardial enzyme/coagulation markers. pANCA/cANCA tests were negative. Ultrasonic cardiogram (UCG) (Figure 2A) revealed biatrial/right ventricular enlargement, left ventricular wall motion abnormalities, reduced LVEF (42%), mild left ventricular wall thickening (4 mm), intraventricular thrombus, and pericardial effusion. After 1 week of respiratory department treatment (anti-infection, LMWH anticoagulation, acid suppression, diuretics), chest CT (Supplementary Figure S1) demonstrated bilateral pulmonary infarctions with consolidations/infiltrates and small pleural effusions. Echocardiography showed further LVEF decline to 36% and rising BNP levels. The patient developed worsening dyspnea, orthopnea, and generalized edema, with unclear ventricular thrombus etiology, prompting transfer to cardiology for further evaluation.

On hospital day 7, genetic testing revealed *MTHFR* C677T (CT genotype) and *PAI-1* 4G/5G polymorphism, indicating mild increased venous thrombosis risk from metabolic/fibrinolytic defects. By day 10, cardiac MRI showed interventricular septal thickening (13 mm), left atrial enlargement, and severe LVEF decline (26.5%). The sputum and pleural fluid was noted to be hemorrhagic (Supplementary Figure S2). Pleural fluid next-generation sequencing identified EBV/CMV, confirming viral myocarditis. Treatment added IV methylprednisolone (100 mg bid), transitioning to oral 1 mg/kg/day with gradual taper. By day 13, the patient reported resolved chest pain, improved edema, and cardiac ultrasound showed septal thickening (15 mm), LVEF 34%, persistent LV thrombus, and normal right heart function (Figure 2B). Despite ongoing anticoagulation, day 14 chest CT revealed progression of pulmonary infarcts, and the patient developed acute right limb weakness (lower limb Muscle strength grade 1) with scattered cerebral infarcts on neuroimaging (Supplementary Figure S3), without major vessel occlusion.

Subsequent testing (day 19) ruled out other respiratory pathogens (Mycoplasma, influenza, SARS-CoV-2). Day 20 cardiac ultrasound showed reduced LV lateral wall, inferoposterior wall, and apical motion, suggesting restrictive myocardial disease. Endomyocardial biopsy revealed focal myocardial vacuolar/hypertrophic degeneration and fibrosis but with no amyloidosis (Supplementary Figure S4). By day 30, left lower lung consolidation with cavitation developed, but overall condition improved with stable vitals and lab trends (Table 1).

By the 33rd day of hospitalization, follow-up ultrasound indicated significant regression of the ventricular thrombus, with a LVEF of 53% and an interventricular septum (IVS) measurement of 11 mm. The patient's symptoms have alleviated, and vital signs are stable, thus meeting the discharge criteria. Post-discharge, the patient is required to continue Warfarin therapy, with weekly monitoring of coagulation INR levels to maintain them within the range of 2.0–3.0.

**Figure 3:**
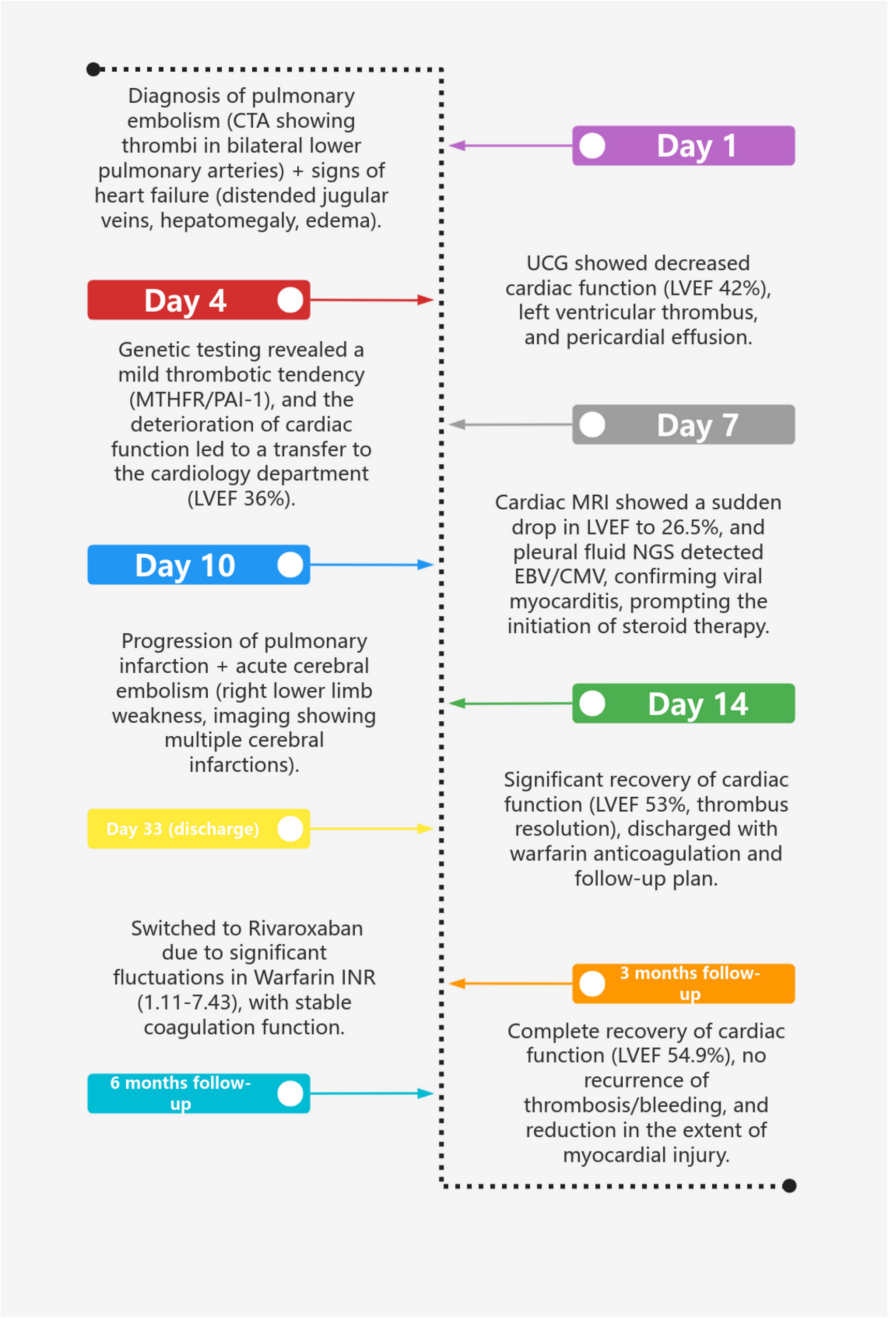
Clinical course timeline.

A follow-up pulmonary CTA should be conducted to assess for pulmonary embolism. Regular follow-ups are necessary to adjust and optimize cardiac function medications, and periodic reviews of blood routine, stool routine with occult blood, liver and kidney function, electrolytes, blood lipids, ECG, and cardiac ultrasound are essential. The patient has been followed up for six months post-discharge and currently reports no discomfort, with stable vital signs. NT-BNP (91.85 pg/ml), Troponin I (TNI) (0.02 ng/ml), suppression of tumorigenicity 2 (ST-2) (7.16 ng/ml), and LVEF (54.9%) are all within normal ranges. Myocardial MR perfusion indicates a reduction in the extent of myocardial injury compared to previous findings. The patient received warfarin anticoagulation therapy after discharge; however, INR levels fluctuated significantly (1.11–7.43), and frequent dose adjustments were unable to maintain stability. In the third month post-discharge, due to poor INR control and an increased risk of bleeding, the treatment was switched to rivaroxaban (20 mg once daily, adjusted based on renal function). After this adjustment, during the three-month follow-up, coagulation function remained stable, with no recurrent thrombotic events or bleeding complications. The clinical course timeline is presented in Figure 3.

## 3 Discussion

Hypercoagulability, a state of heightened thrombotic risk, is well-characterized in SARS-CoV-2 infection but less explored in EBV and CMV infections. This case illustrates how EBV/CMV-mediated myocardial injury initiates a vicious cycle of pathophysiology, highlighting diagnostic challenges and therapeutic considerations.

EBV and CMV directly invade myocardial cells, triggering cellular damage. CMV impairs mitochondrial function, leading to energy metabolism dysfunction and necroptosis (6). Myocarditis induced by EBV consists of three separate phases: the acute stage defined by the activation of the innate immune system, the subacute stage distinguished by the activation of the adaptive immune response, and the phase of chronic inflammation. Throughout these phases, both activated innate immune cells and cardiac cells secrete a range of cytokines, chemokines, interferons, and alarmins. This secretion promotes additional activation and recruitment of innate immune cells to the heart, encompassing mast cells, neutrophils, dendritic cells, monocytes, and macrophages (7). Inflammatory cascades further drive coagulation. TNF-α induces angiotensin II (AngⅡ) accumulation via ADAM17-mediated ACE2 shedding, upregulating endothelial tissue factor (TF) expression (8). Interleukin-6 (IL-6) disrupts vascular barriers through JAK/STAT3 signaling, enhancing fibrinogen extravasation and platelet aggregation (9). This creates a procoagulant “inflammation-coagulation” feedback loop, analogous to COVID-19 (10). In addition, direct exposure of procoagulant substances due to endothelial injury, blood stasis that creates an environment conducive to thrombus formation (11). Moreover, the immune evasion mechanisms employed by the virus further exacerbate myocardial injury. TRIM29, an E3 ubiquitin ligase responsive to viral stimuli, facilitates persistent EBV infection by inhibiting the production of type I interferons (12). Recent studies have demonstrated that TRIM29 is significantly induced by cardiac viruses, promoting protein kinase RNA-like endoplasmic reticulum kinase (PERK)-mediated endoplasmic reticulum (ER) stress, cell apoptosis, and reactive oxygen species (ROS) responses. These processes facilitate viral replication in cardiomyocytes *in vitro* (13). In clinical practice, the dynamic expression of TRIM29 may serve as a novel biomarker. Future studies are necessary to validate the diagnostic value of TRIM29 in comparison to traditional markers such as troponin and LVEF.

The *MTHFR* (C677T) mutation reduces enzyme activity (30%–65% of wild type), decreasing 5-methyltetrahydrofolate and increasing homocysteine (Hcy), leading to endothelial injury via oxidative/inflammatory/endoplasmic reticulum stress (14–16). Elevated Hcy enhances platelet aggregation and impairs fibrinolysis (17, 18). TT genotype patients require high-dose folic acid (0.8–5 mg/d) + vitamin B12 to reduce Hcy by 30%–50% and stroke recurrence, while CC/CT genotypes respond to conventional doses (0.4 mg/d) (19). The *PAI-1* 4G/5G promoter polymorphism is associated with altered plasma PAI-1 levels (20, 21). A study from China has validated through molecular detection techniques and clinical cohort analysis that the *PAI-1* 4G/5G polymorphism serves as a prognostic predictor rather than a susceptibility factor in Chinese VTE patients, confirming its core clinical value (22).

In patients with multi-site embolism, comprehensive thrombophilia screening is critical. Inherited causes (e.g., antithrombin/protein C/S deficiency, Factor V Leiden, prothrombin G20210A) and acquired factors (e.g., antiphospholipid syndrome, malignancy, immobilization) must be evaluated. Although relevant laboratory tests were conducted upon admission in this case, they could only provide preliminary insights into the coagulation and inflammatory status, without confirming or excluding thrombophilia. Genetic testing indicated the presence of mutations in *MTHFR* (C677T) and *PAI-1* (4G/5G). Further assessments are necessary for antithrombin, protein C, protein S, factor V Leiden mutation, and prothrombin G20210A mutation. Regarding the exclusion of acquired thrombophilia, the patient has no history of malignancies, and tests for common respiratory pathogens and autoimmune-related indicators returned negative results, which somewhat rules out thrombophilia linked to infections and autoimmune diseases. However, the patient's 20-year history of hypertension poses a risk for vascular endothelial injury, and prolonged bed rest following the illness are both factors that may contribute to thrombosis. In summary, the current examinations have screened only a subset of thrombophilia factors. To establish a definitive diagnosis, it is imperative to complete additional tests and conduct a comprehensive assessment of thrombophilia risk, thereby facilitating the precise formulation of treatment strategies to mitigate the risk of thrombosis recurrence.

Myocardial biopsy, a key diagnostic tool, is often delayed due to hemodynamic instability or invasiveness. Hormone therapy may mask inflammatory pathology, complicating diagnosis. Early etiological (EBV/CMV) and genetic screening are critical for targeted treatment. Delay in antiviral therapy (for EBV/CMV) or folate/B12 supplementation (for *MTHFR*) can worsen outcomes. Proactive biopsy and multi-specimen next-generation sequencing [NGS; e.g., blood, bronchoalveolar lavage fluid (BALF), myocardial tissue] enhance pathogen detection specificity, while immunohistochemistry/*in situ* hybridization localizes viral antigens/nucleic acids. Serial monitoring of CMV/EBV DNA loads aids in confirming active infection.

In the case of a patient with multiple-site embolism, genetic fibrinolytic disorder (specifically PAI-1 4G/5G and *MTHFR* C677T mutations), and unique characteristics of anticoagulation therapy, it is crucial to prioritize early screening for viral infections (EBV/CMV) and hypercoagulable-related genetic factors to elucidate the etiology and thrombotic propensity. Risk stratification should be conducted utilizing the sPESI score for low-to-intermediate risk pulmonary embolism and assessing LVEF to evaluate the risk of left ventricular thrombus. We initially chose warfarin for anticoagulation upon the patient's discharge is primarily based on the following considerations: (1) The patient exhibits the *PAI-1* 4G/5G and *MTHFR* C677T mutations, necessitating long-term anticoagulation to prevent recurrence. As a traditional oral anticoagulant, warfarin has substantial evidence-based support for its use in patients with thrombophilia (23); (2) The complexity of multi-site thrombosis is exemplified by the simultaneous occurrence of pulmonary embolism, left ventricular thrombus, and cerebral infarction. Warfarin facilitates flexible management of varying thrombotic risks through adjustments in the INR (24, 25). Anticoagulant therapy should be adjusted according to body weight, administering enoxaparin at a dosage of 1 mg/kg every 12 h, while warfarin requires a higher initial dose (5–7.5 mg/day) due to the *MTHFR* mutation, necessitating INR monitoring within the therapeutic range of 2.0–3.0. DOACs should be prescribed with caution, considering the increased bleeding risk in Asian populations and renal function. Furthermore, anticoagulation should be deferred following cerebral infarction until the acute phase has resolved, with vigilant monitoring for signs of bleeding, including fecal occult blood and cranial CT imaging. After discharge, the patient exhibited unstable INR levels while on warfarin, leading to a transition to rivaroxaban three months after discharge, during which no thrombotic events were noted. The rationale for selecting rivaroxaban was its fixed-dose regimen, which eliminates the need for INR monitoring and thereby reduces the risk of bleeding events associated with INR fluctuations (26). Importantly, no recurrence of thrombosis or bleeding complications was observed within three months following the switch, confirming the safety of this treatment. For patients with hereditary thrombophilia and a documented history of thrombosis, long-term or even lifelong anticoagulation therapy is essential to prevent recurrence.

For patients with thrombophilia and inadequate INR control, NOACs may provide a viable alternative to warfarin. In clinical practice, it is imperative to optimize dosing, supplement with folate and vitamin B12 to correct hyperhomocysteinemia, and engage in multidisciplinary follow-up assessments involving cardiology, hematology, and neurology to evaluate thrombus resolution and bleeding risk.

In this case, although the NGS of pleural fluid indicated the coexistence of CMV and EBV, it was not possible to determine which pathogen was primarily responsible for the myocarditis. Therefore, early multi-specimen testing is recommended for such patients. In addition to pleural fluid, it is advisable to simultaneously collect myocardial biopsy tissue, blood, and BALF for NGS to enhance the specificity of pathogen detection. Furthermore, immunohistochemical testing should be performed to detect EBV/CMV antigen expression in myocardial tissue, or *in situ* hybridization can be utilized to localize viral nucleic acids. Continuous monitoring of plasma EBV/CMV-DNA load is essential, as a sustained increase in viral load may support the diagnosis of active infection.

## 4 Conclusion

This case illustrates the intricate pathological mechanism of multi-organ embolism resulting from EBV/CMV infection in conjunction with hereditary thrombophilia, specifically the *MTHFR* C677T and *PAI-1* 4G/5G mutations. EBV and CMV activate the coagulation system through direct myocardial injury and a cytokine storm. This process is compounded by the fibrinolytic inhibition and hyperhomocysteinemia induced by genetic mutations, creating a vicious cycle of synergistic thrombosis characterized by “virus-inflammation-gene” interactions. In clinical practice, early viral detection methods, such as myocardial biopsy and genetic screening, are essential for elucidating the etiology. Furthermore, personalized anticoagulation strategies, transitioning from enoxaparin to warfarin and then to rivaroxaban, combined with multidisciplinary management, effectively balance the risks of thrombosis and bleeding.

## Data Availability

The raw data supporting the conclusions of this article will be made available by the authors, without undue reservation.

## Supplementary material

Supplementary Figure S1 Chest CT demonstrated bilateral pulmonary infarctions with consolidations/infiltrates and small pleural effusions.

Supplementary Figure S2 Putum and pleural fluid were observed to be hemorrhagic.

Supplementary Figure S3 A CT scan and MR perfusion of the head were performed, which indicated multiple scattered acute cerebral infarcts in both cerebral hemispheres.

Supplementary Figure S4 The myocardial biopsy showed partial vacuolar degeneration of myocardial cells, slight hypertrophic degeneration, focal fibrosis, and no evidence of amyloidosis.
