# Supplementary figures and images for: When viral myocarditis meets thrombosis tendency: deep analysis of a complex case report
Source: Front Cardiovasc Med. 2025 Aug 20;12:1641074. doi: 10.3389/fcvm.2025.1641074 (PMC12405257; doi:10.3389/fcvm.2025.1641074)

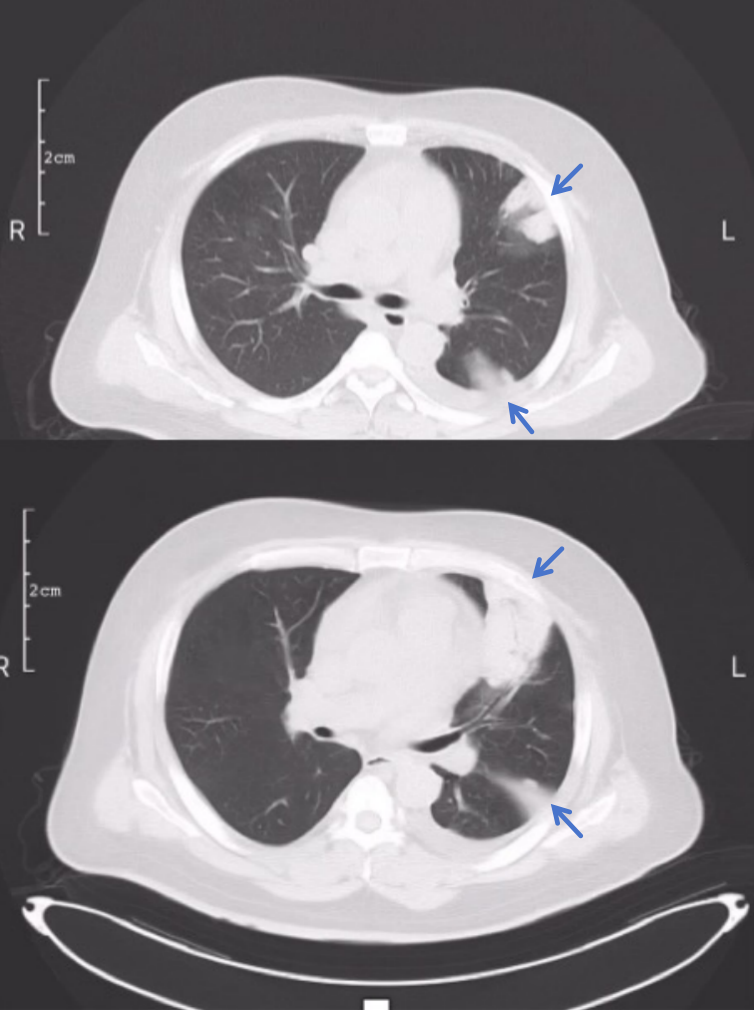

Supplement: Supplementary Figure S1 — Chest CT demonstrated bilateral pulmonary infarctions with consolidations/infiltrates and small pleural effusions. [file Image1.jpeg]

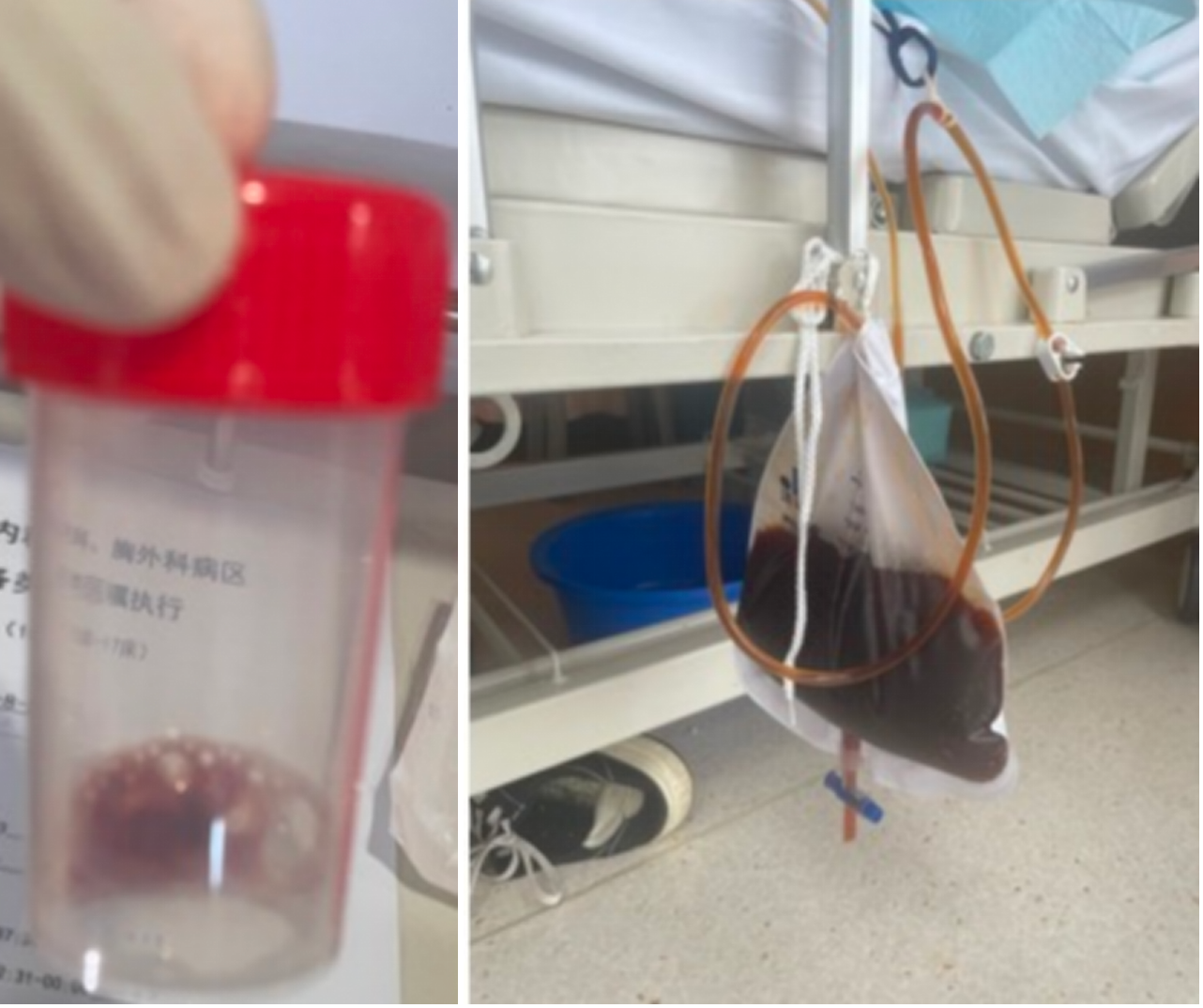

Supplement: Supplementary Figure S2 — Putum and pleural fluid were observed to be hemorrhagic. [file Image2.jpeg]

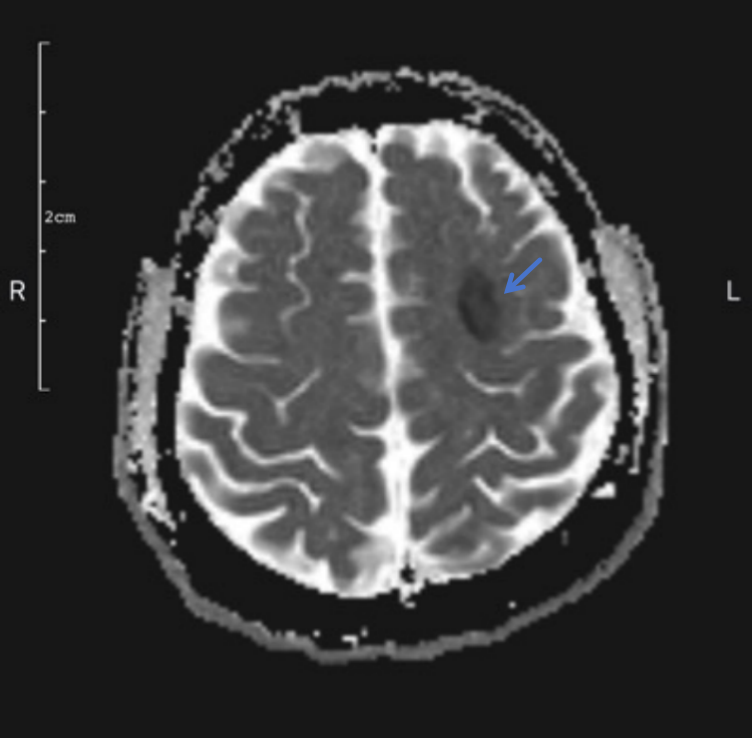

Supplement: Supplementary Figure S3 — A CT scan and MR perfusion of the head were performed, which indicated multiple scattered acute cerebral infarcts in both cerebral hemispheres. [file Image3.jpeg]

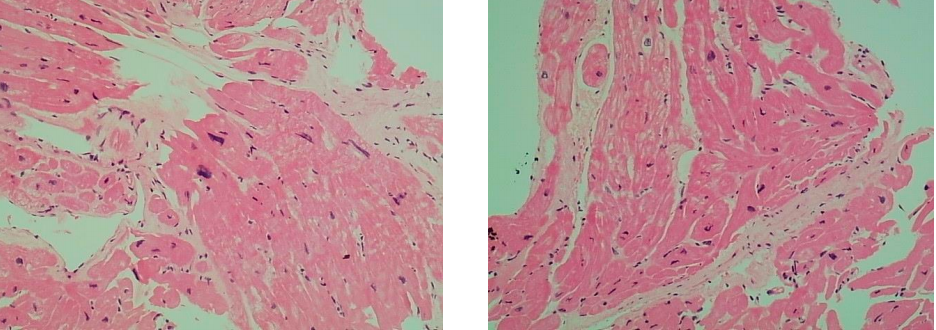

Supplement: Supplementary Figure S4 — The myocardial biopsy showed partial vacuolar degeneration of myocardial cells, slight hypertrophic degeneration, focal fibrosis, and no evidence of amyloidosis. [file Image4.jpeg]
